# Cyanidin-3-o-glucoside directly binds to ERα36 and inhibits EGFR-positive triple-negative breast cancer

**DOI:** 10.18632/oncotarget.12025

**Published:** 2016-09-15

**Authors:** Li Wang, Haifeng Li, Shiping Yang, Wenqiang Ma, Mei Liu, Shichao Guo, Jun Zhan, Hongquan Zhang, Suk Ying Tsang, Ziding Zhang, Zhaoyi Wang, Xiru Li, Yang-Dong Guo, Xiangdong Li

**Affiliations:** ^1^ State Key Laboratory of the Agro-Biotechnology, College of Horticultural Science, China Agricultural University, Beijing, China; ^2^ Department of General Surgery, The 301th Hospital of PLA, Beijing, China; ^3^ Key Laboratory of Carcinogenesis and Translational Research, Ministry of Education, State Key Laboratory of Natural and Biomimetic Drugs, Peking University Health Science Center, Beijing, China; ^4^ School of Life Sciences and State Key Laboratory of Agro-Biotechnology, Chinese University of Hong Kong, Hong Kong, China; ^5^ Beijing Shenogen Pharma Group, Beijing, China

**Keywords:** Cy-3-glu, ERα36, EGFR, triple-negative breast cancer, apoptosis

## Abstract

Anthocyanins have been shown to inhibit the growth and metastatic potential of breast cancer (BC) cells. However, the effects of individual anthocyanins on triple-negative breast cancer (TNBC) have not yet been studied. In this study, we found that cyanidin-3-o-glucoside (Cy-3-glu) preferentially promotes the apoptosis of TNBC cells, which co-express the estrogen receptor alpha 36 (ERα36) and the epidermal growth factor receptor (EGFR). We demonstrated that Cy-3-glu directly binds to the ligand-binding domain (LBD) of ERα36, inhibits EGFR/AKT signaling, and promotes EGFR degradation. We also confirmed the therapeutic efficacy of Cy-3-glu on TNBC in the xenograft mouse model. Our data indicates that Cy-3-glu could be a novel preventive/therapeutic agent against the TNBC co-expressed ERα36/EGFR.

## INTRODUCTION

Triple-negative breast cancer (TNBC), one of multiple clinical subtypes of breast cancer (BC) defined by a lack of expression of estrogen receptors (ER), progesterone receptors (PR), and the HER-2/neu epidermal growth factor receptors [1], accounts for 10-15% of all BC cases [2]. TNBC often responds poorly to available chemotherapies [3]. Due to the lack of preclinical biomarkers for this subtype of BC, the suitable clinical strategies for its treatment and prevention are barely defined. Accordingly, the identification of specific and effective molecular targets for effective therapies in TNBC is an urgent and unmet need.

TNBC is sometimes classified into a “basal-type” cancer group, which is frequently defined by cytokeratin 5/6 and epidermal growth factor receptor (EGFR) positive staining. Approximately 75% of basal-type BCs are TNBC. The overexpression of EGFR has been demonstrated in up to 88.5-89.5% of TNBC patients [4, 5] since EGFR was identified as a therapeutic target for TNBC patients in 2011 [6]. Nevertheless, no clear criteria have been standardized yet [7].

Although TNBC is characterized by a lack of ERα expression, several studies showed that an ovariectomy did prevent the progression of TNBC. A case-control study of 187 TNBC cases described a 2.5 increased risk for women who used oral contraceptives (OCs) for more than one year compared to women who used OCs for less than one year or never [8]. Altogether, these data implied that estrogen still plays a critical role in the etiology of TNBC. The ERα36, a 36-kDa variant of ERα, is highly expressed in TNBC [9] and has been shown to be involve in membrane-initiated and rapid estrogen signaling [10]. EGFR was found to have therapeutic effects on TNBC and is now undergoing preclinical/clinical investigations [11]. These studies strongly emphasized the causal link between ERα36 and the EGFR signaling pathway in the etiology of TNBC [9].

Anthocyanins belong to a group of molecules called flavonoids, are derived from anthocyanidins, contribute to the intense color of many fruits, vegetables, and pigments, and are abundant in our daily diet [12]. There are six particularly important anthocyanidins, including cyanidin, delphinidin, pelargonidin, malvidin, peonidin and petunidin. Due to their instabilities in nature, acylated anthocyanidins are most frequently produced and glycosylated 2 or 3-fold with monosaccharides [13] to form anthocyanins. Anti-oxidant [14], anti-inflammatory [15], and anti-proliferative [16, 17] activities for a mixture of anthocyanins have been reported. However, the anti-TNBC effects of the individual anthocyanins have not been well studied. As Cy-3-glu is the most abundant anthocyanin pigment in many vegetables and fruits [18], the goal of this study was to figure out the mechanism mediating the effects of Cy-3-glu in the prevention of TNBC.

## RESULTS

### Cy-3-glu inhibits the growth of TNBC cells

**Figure 1:**
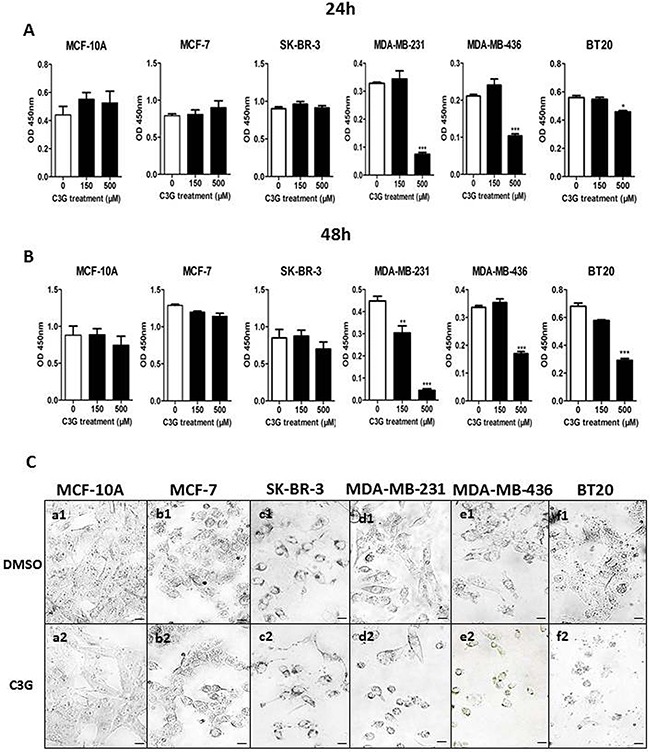
Cy-3-glu inhibits the growth of TNBC cells **A** and **B.** the prolifereation of cells analyzed by the CCK-8 assay. Cells were treated with 150 μM and 500 μM Cy-3-glu for 24 h and 48 h; DMSO serves as the vehicle control. **C.** cell morphology observed by phase-contrast microscopy corresponding to Figure 1B. The upper panel (a1 to f1) shows the controls, and the lower panel (a2 to f2) shows the Cy-3-glutreated cells after 48 h. The results represent the mean ± SEM from three independent experiments. Differences with p < 0.05 (*), p < 0.01 (**) or p < 0.001(***) were considered statistically significant. Scale bar is 100 μm.

Cy-3-glu is the most widespread glycoside class of anthocyanin pigments in vegetables and fruits (Supplementary Table S1) [19]; the contents of Cy-3-glu is usually more than 10-100 times higher than delphinidin-3-glucoside, petunidin-3-glucoside, peonidin-3-glucoside, pelargonidin-3-glucoside and malvidin-3-glucoside. To verify the efficacy of Cy-3-glu against BC, several cell lines representing different clinical subtypes of BC were examined. We treated MDA-MB-231 cells with low dose Cy-3-glu (5 and 10 μM) for a longer time (7 d) and found that the growth of MDA-MB-231 cells was significantly inhibited (p = 0.022) at 7 d (Supplementary Figure S1A). In order to study the mechanism and shorten the experiment time, we use the higher doses of Cy-3-glu (150 and 500 μM) and set up the time points at 24 and 48 h, respectively. We observed no significantly additional cytotoxicity at 48 h with higher dose (150 and 500 μM) of Cy-3-glu treatment (Supplementary Figure S1B). Treatment with both doses, the 150 and 500 μM of Cy-3-glu, significantly inhibited TNBC cell growth, as shown in Figure 1. Specifically, MDA-MB-231 (p < 0.001 at 48 h), MDA-MB-436 (p < 0.001 at 24 h and 48 h; 500 μM) and BT20 cells (p < 0.5 at 24 h and p < 0.001 at 48 h; 500 μM). Actually, a greater growth inhibitory effect was observed on MDA-MB-231 cells (Figure 1A and 1B), and this may due to different sensitivities of individual cell lines. In comparison, no significant changes were obtained in the non-TNBC cells, including the MCF-7, SK-BR-3 and the non-cancerous breast epithelial cells MCF-10A treated with 500 μM Cy-3-glu for 24 h and 48 h (Figure 1A and 1B). Morphological analysis of the cells also showed a significant change in the MDA-MB-231, MDA-MB-436 and BT20 cells, which were characterized by decreased cell volume, concomitant shrinking, pyknotic nucleus and foaming of the cell membrane. In comparison, the MCF-7, SK-BR-3 and MCF-10A cell lines did not show any of the characteristic features observed in the TNBC after the 48 h treatment (Figure 1C). Taken together, the data demonstrated that Cy-3-glu inhibits TNBC cell growth more effectively compared to non-TNBC cells, and has no effect on non-cancerous breast epithelial cells.

### Cy-3-glu induces TNBC MDA-MB-231 cell apoptosis with no effect on the cell-cycle

**Figure 2:**
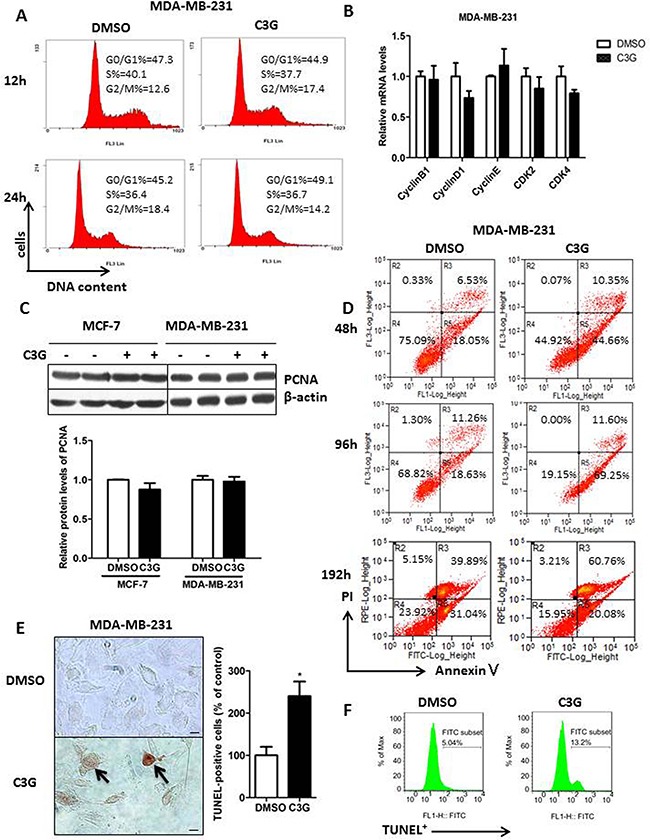
Cy-3-glu induces apoptosis in the MDA-MB-231 TNBC cells without affecting the cell-cycle **A.** Evaluation of Cy-3-glu effects on MDA-MB-231 cell cycle progression by flow cytometry after 12 h and 24 h treatments. DNA content was measured by PI staining (x axis) and the population of cells was measured (y axis). **B.** relative mRNA expression levels of *cyclin B1, cyclin D1, cyclin E, CDK2* and *CDK4* after treatment with Cy-3-glu (150 μM) for 24 h in MDA-MB-231 cells. **C.** upper panel, immunoblots of PCNA in MCF-7 and MDA-MB-231 cells untreated or treated with Cy-3-glu; β-actin was used as the internal control. Lower panel, bar graphs show the relative protein levels of PCNA. **D.** Annexin V/PI double staining for apoptotic MDA-MB-231 cells by flow cytometry at 48 h, 96 h, and 192 h Cy-3-glu treatment. Phosphatidylserine was measured by annexin V–FITC staining (x axis), and apoptosis was measured by PI staining (y axis). **E.** left panel, TUNEL staining to detect late apoptotic cells after Cy-3-glu treatment. Late apoptotic cells had brownish nuclear regions (arrows). Right panel, bar graphs show the number of TUNEL-positive cells. **F.** TUNEL staining for apoptotic MDA-MB-231 cells after Cy-3-glu treatment for 48 h by flow cytometer, and the determined percentage of TUNEL^+^ cells. The results represent the mean ± SEM from three independent experiments. Scale bar is 100 μm.

We next asked whether the suppression of cell growth by Cy-3-glu was due to any effect on the cell-cycle. To evaluate the effects of Cy-3-glu on the cell cycle, flow cytometry analysis was performed on MDA-MB-231 cells. We observed no alterations in the sub-G0/G1 phase, S phase and G2/M population after Cy-3-glu treatment for 12, 24 and 48 h (Figure 2A and Supplementary Figure S2). We also found no significant changes in the mRNA expression of cell cycle regulatory molecules (*cyclinB1, cyclinD1, cyclinE, CDK2 and CDK4*) after Cy-3-glu treatment for 24 h (Figure 2B). In addition, we detected no changes in the proliferating cell nuclear antigen (PCNA) by Western blot analysis (Figure 2C). These results indicated that Cy-3-glu inhibited MDA-MB-231 cell growth independent of cell-cycle arrest. However, quantitation of flow cytometric analysis results demonstrated that Cy-3-glu treatments (48, 96 and 192 h) induced a dramatic increase in the amount of apoptotic cells in the experiment with MDA-MB-231 cells along the time (Figure 2D). The TUNEL assay also showed an increased number of dead cells (Figure 2E) and positive cells from 5.04% to 13.20% by flow cytometry (Figure 2F) after Cy-3-glu treatment of MDA-MB-231 cells. In summary, these results indicated that Cy-3-glu inhibited growth of MDA-MB-231 cells by inducing apoptotic death rather than cell-cycle arrest.

### Cy-3-glu induces TNBC MDA-MB-231 cell death by apoptosis, not necroptosis, by up caspases cascade pathway

**Figure 3:**
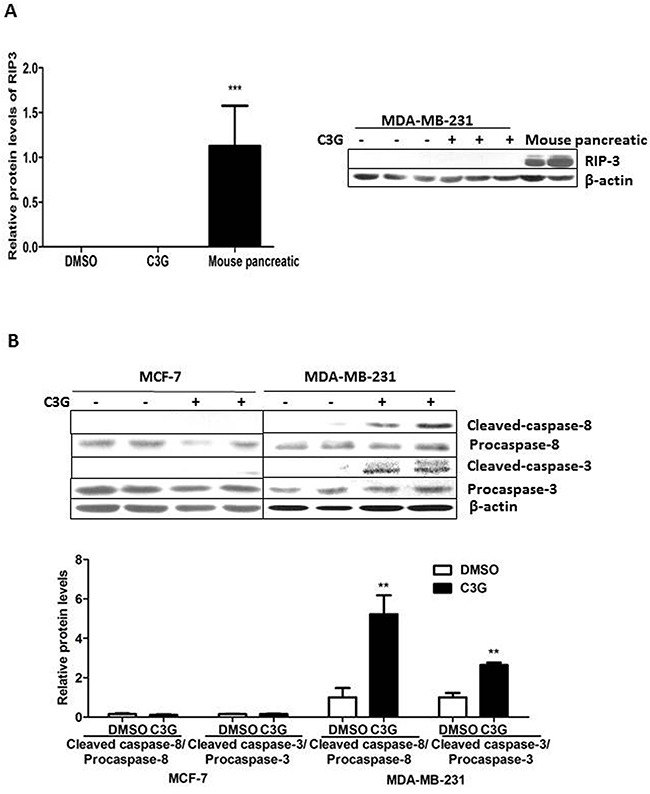
Cy-3-glu induces TNBC MDA-MB-231 cell death by the extrinsic apoptosis pathway, not necroptosis or by the intrinsic pathway MCF-7 and MDA-MB-231 cells were treated with Cy-3-glu (150 μM) for 24 h in FBS-free cell culture medium. **A.** left panel, bar graphs show the relative protein levels of RIP3. Right panel, RIP3 expression in MDA-MB-231 detected by Western blotting; the mouse pancreas was used as the positive control. **B.** upper panel, expression levels of cleaved-caspase-3 (17 kDa and 19 kDa), cleaved-caspase-8 (47 kDa), caspase-3 (35 kDa) and caspase-8 (57 kDa). β-actin was the internal control. Lower panel shows the relative protein levels of cleaved-caspase-8 and cleaved-caspase-3 in MCF-7 and MDA-MB-231 cells. The results represent the mean ± SEM from three independent experiments. Differences with p < 0.05 (*), p < 0.01 (**) or p < 0.001(***) are considered statistically significant.

Since both apoptosis [20] and necroptosis [21] are considered two of the important mechanisms of BC cell death. We first evaluated RIP3 expression in MDA-MB-231 cells, using the mouse pancreas as a positive control (Figure 3A). Our failure to detect RIP3 expression in MDA-MB-231 cells treated with Cy-3-glu, suggested that necroptosis was not involved in Cy-3-glu-induced cells death. Apoptosis mediated by the TNF cytokine is referred to as the extrinsic apoptosis pathway and is initiated by the binding of TNF to its cell surface receptors (TNFRs) thus activating the initiator caspase-8, followed by activation of the downstream effector caspase-3, resulting in cleavage of critical cellular proteins, ultimately leading to cell death [22]. Indeed, we observed an increase in cleaved-caspase-8 after Cy-3-glu treatment, and consequently an increase of cleaved-caspase-3 in MDA-MB-231 cells. On the other hand, no detectable cleaved-caspase-8 or cleaved-caspase-3 was observed in MCF-7 cells (Figure 3B). Together these results show that Cy-3-glu promoted cell apoptosis through activation of the caspases cascade in MDA-MB-231, but not in MCF-7 cells.

### Cy-3-glu induces TNBC MDA-MB-231 apoptosis via the non-mitochondrial pathway

**Figure 4:**
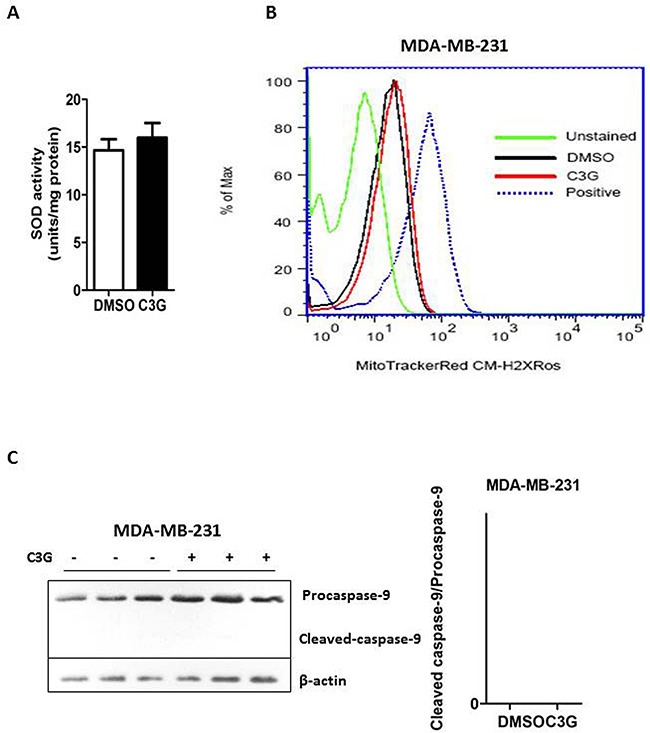
Cy-3-glu induces apoptosis of the MDA-MB-231 TNBC cells via the non-mitochondrial (extrinsic) pathway Cells were treated with Cy-3-glu (500 μM) for 24 h. **A.** assessment of SOD activity between the DMSO and Cy-3-glu-treated groups. **B.** 30 min after the addition of CM-H2XRos to MDA-MB-231 cells, mitochondrial ROS fluorescent images were captured by flow cytometry. Mitochondrial ROS in MDA-MB-231 cells was stained by MitoTrackerRed CM-H2XRos. Non-stained groups were unstained and positive groups were treated with H_2_O_2_ before staining by MitoTrackerRed CM-H2XRos. X axis is the CM-H2XRos staining and y axis indicates the percentage of MDA-MB-231 cell populations between DMSO and Cy-3-glu treatments. **C.** left panel, the expression of caspase-9 and cleaved-caspase-9 in MDA-MB-231 cells was detected by Western blotting. β-actin was used as the internal control. Right panel, bar graph shows the relative protein levels of cleaved-caspase-9/caspase-9. Data are represented as the mean ± SEM (n = 3). Triplicate measurements were performed for each experiment.

Superoxide dismutase (SOD), an important indicator of anti-oxidant capacity, reduces the superoxide anion to maintain the cellular redox homeostasis [23]. We determined the cellular SOD activity after Cy-3-glu treatment and found no significant changes in MDA-MB-231 cells (Figure 4A, p = 0.530). Mitochondria are a key player in apoptosis through the generation of reactive oxygen species (ROS) and release of cytochrome C and the subsequent activation of caspase-9 and caspase-3/7 [24–26]. However, in our study we observed no significant mitochondrial function changes between MDA-MB-231 cells treated with DMSO or Cy-3-glu (Figure 4B). In addition, we failed to detect the expression of active caspase-9 (cleaved-caspase-9) in MDA-MB-231 cells (Figure 4C). These results indicated that Cy-3-glu induces MDA-MB-231 cell apoptosis independent of the mitochondrial intrinsic pathway.

### Cy-3-glu counteracts estrogen-induced proliferation of TNBC cells

**Figure 5:**
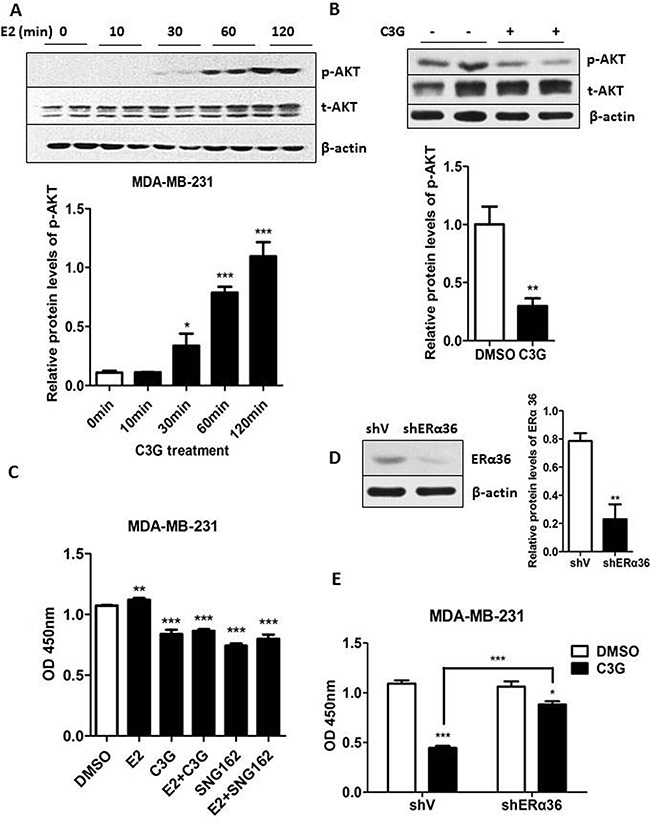
Cy-3-glu counteracts the estrogen-induced proliferation of MDA-MB-231 TNBC cells **A.** MDA-MB-231 cells were treated with E2 (100 nM) for the indicated time periods in FBS-free cell culture medium. The intensity of each band was quantified, and expressed as a percentage using the time 0 as reference. Upper panel, the expression of phosphorylated-AKT (p-AKT) and total AKT (t-AKT) were determined after E2 treatment. Lower panel, bar graphs show the relative protein levels of p-AKT/t-AKT. **B.** upper panel, the expression of t-AKT and p-AKT after Cy-3-glu (150 μM) treatment was determined. β-actin was used as the internal control. Lower panel shows the ratios of p-AKT/t-AKT. **C.** MDA-MB-231 cells proliferation was analyzed by the CCK-8 assay at 24 h. Cells were treated with 1 nM E2 and 500 μM Cy-3-glu, or E2 plus Cy-3-glu, or ERα36 inhibitor SNG162 or E2 plus ERα36 inhibitor SNG162, DMSO serves as the vehicle control. **D.** left panel, Western blot analysis of MDA-MB-231 cells transfected with a control vector (shV) and with the ER-α36 shRNA vector (shERα36). Right panel, bar graphs show the relative protein levels of ERα36. **E.** the proliferation of Cy-3-glu-induced MDA-MB-231 cells was analyzed after knock down of ERα36 by the shRNA method. The immunoblots shown here are from a representative experiment repeated three times with similar results. Differences with p < 0.05 (*), p < 0.01 (**) or p < 0.001(***) are considered statistically significant.

Due to lack of ERα expression, a prevailing view is that estrogen signaling is not involved in the development and progression of ER-negative BC. However, consistent with a previous report [27], in the present study E2 induced phosphorylation of the AKT in MDA-MB-231 cells at different time periods. The results shown in Figure 5A indicate that AKT phosphorylation occurred within 30 min after E2 application and sustained the activation for up to 120 min. Additionally, we also found that Cy-3-glu decreased the level of p-AKT (Figure 5B) and inhibited estrogen-stimulated proliferation of MDA-MB-231 cells (Figure 5C). Actually, previous studies indicated that ERα36 is highly expressed in MDA-MB-231 cells, with lower levels found in ER-positive MCF-7 cells, and it is undetectable in the mammary epithelial cells MCF-10A [9, 10]. We found that SNG162 significantly inhibited the growth of MDA-MB-231 cells stimulated by E2 as a positive control (Figure 5C). In addition, the knocked down of ERα36 by shRNA in MDA-MB-231 cells (Figure 5D) revealed that the knocking down of ERα36 could significant counteract the growth inhibitory effect of Cy-3-glu on MDA-MB-231 cells (Figure 5E). From these results we inferred that Cy-3-glu inhibits the growth of MDA-MB-231 cells, presumably through ERα36.

### Cy-3-glu binds to the LBD of ERα36 directly and inhibits its signaling pathway in TNBC cells

**Figure 6:**
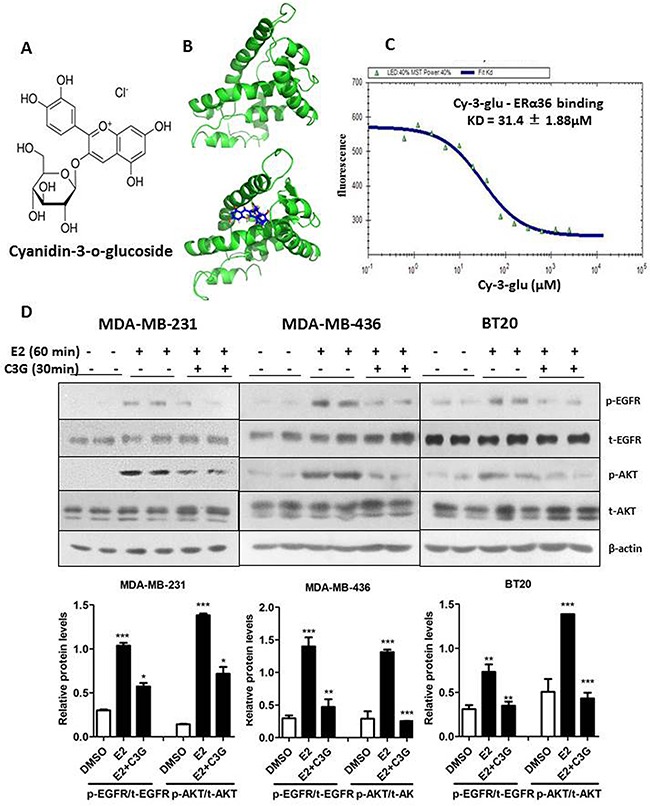
Cy-3-glu binds to LBD of ERα36 directly and inhibits its signaling pathway in TNBC cells **A.** the chemical structure of Cy-3-glu. **B.** upper panel, a simulated docking model of the LBD-ERα36 (green color) complex. Lower panel, a simulated docking model of Cy-3-glu (dark blue color) in the pocket of LBD-ERα36 (green color). **C.** microscale thermophoresis analysis of Cy-3-glu binding to LBD-ERα36. Purified LBD-ERα36 protein was first labeled with Cy5/Alexa 647 fluorescence dye. Cy-3-glu was titrated between 0.61 and 2500 μM to the constant amount of labeled proteins (100 nM) and the binding affinity is 31.4 ± 1.88 μM. **D.** upper panel, phosphorylated-EGFR (p-EGFR), total EGFR (t-EGFR), p-AKT and t-AKT in TNBC cells were detected by Western blotting. Cy-3-glu (150 μM) for 30 min before E2 (100 nM) for 60 min. MDA-MB-231, MDA-MB-436 and BT20 cells were harvested and lysed. Lower panel, bar graphs show the relative protein levels of p-EGFR/t-EGFR and p-AKT/t-AKT. The immunoblots shown here are from a representative experiment repeated three times with similar results. Differences with p < 0.05 (*), p < 0.01 (**) or p < 0.001(***) are considered statistically significant.

To evaluate the binding affinity between Cy-3-glu and the LBD of ERα36, we first conducted molecular docking analysis using the Auto Dock program (www.scripps.edu/mb/olson/doc/autodock). The conformation is derived from co-crystallization data of the ERα66 conformation (Protein Data Bank Code: 2YJA). By docking the conformation of LBD-ERα36 with Cy-3-glu (Figure 6B) and merging the conformations of LBD-ERα36 and LBD-ERα66 (Supplementary Figure S3A), we observed there are deletions of α8 and α9 helixes in ERα36 compare to ERα66, which lead to a more open binding pocket of ERα36. Molecular docking studies showed that Cy-3-glu had a stronger binding affinity score with ERα36 (binding energy is -79.89 kcal/mol) (Supplementary Figure S3B) than with ERα66 (binding energy is -28.27 kcal/mol) (Supplementary Figure S3C). Accordingly, compare to ERα66, Cy-3-glu was more stable and easier to fit into the ligand-binding pocket of ERα36. Furthermore, we checked the binding constant of Cy-3-glu to LBD-ERα36 and LBD-ERα66 by MST analysis, which allows for the sensitive detection of small-molecule binding to a protein target [28]. The data showed that Cy-3-glu binds to LBD-ERα36 with a KD of 31.4 ± 1.88 μM (Figure 6C) and almost no binding to LBD of ERα66 were observed compared to E2 positive control (Supplementary Figure S4A and S4B). We have previously reported that EGFR is one of the most important downstream targets of activated ERα36 signaling [9]. Thus, to further determine the effects of Cy-3-glu on ERα36 signaling pathway, we analyzed the levels of EGFR and AKT phosphorylation. The analysis revealed that Cy-3-glu significantly inhibited the E2-induced phosphorylation levels of the EGFR and AKT in TNBC MDA-MB-231, MDA-MB-436 and BT20 cells (Figure 6D). In summary, our data demonstrated that Cy-3-glu directly binds to the ERα36 receptor and, in turn, inhibits its downstream signaling.

### The binding of Cy-3-glu to the LBD of ERα36 leads to the degradation of EGFR through the proteasome system in TNBC cells

**Figure 7:**
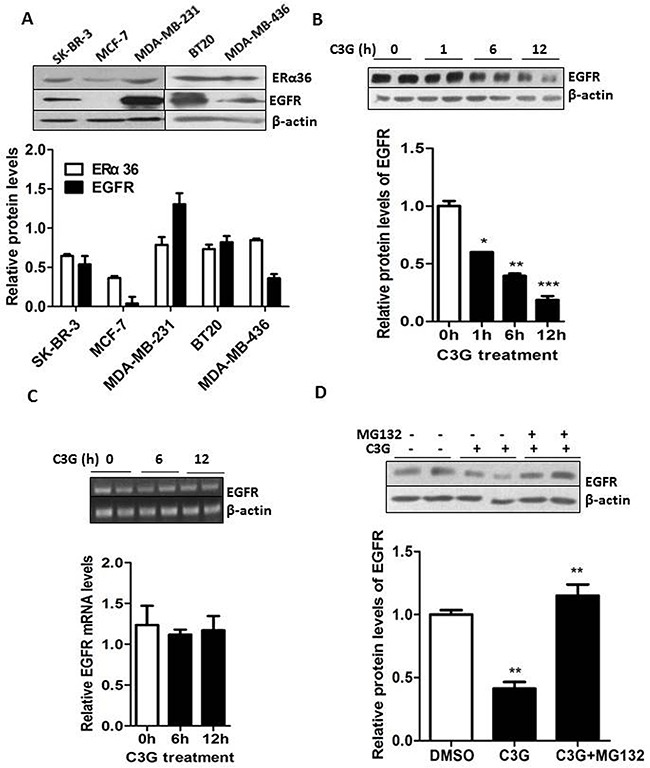
Binding of Cy-3-glu to the LBD of ERα36 leads to degradation of EGFR through the proteasome system in MDA-MB-231 TNBC cells **A.** upper panel, the expression of ERα36 and EGFR in several BC cell lines. Lower panel, bar graphs show the relative protein levels of ERα36 and EGFR. **B.** MDA-MB-231 cells were treated with Cy-3-glu (150 μM) for 0 h, 1 h, 6 h and 12 h in FBS-free cell medium. Upper panel, the expression of EGFR was determined after Cy-3-glu treatment. Lower panel, bar graphs show the relative protein levels of EGFR. **C.** upper panel, RT-PCR analysis of *EGFR* mRNA levels after Cy-3-glu (150 μM) treatment at 0 h, 6 h and 12 h. Lower panel, bar graphs show the relative mRNA levels of *EGFR*. **D.** upper panel, EGFR degradation through the proteasome system was detected by Western blotting. MG132 was added 30 min before treatment with Cy-3-glu for 6 h. The intensity of each band was quantified, and expressed as the percentage using the time 0 h as reference. Lower panel, bar graphs show the relative protein levels of EGFR. The immunoblots shown here are from a representative experiment repeated three times with similar results. Differences with p < 0.05 (*), p < 0.01 (**) or p < 0.001(***) are considered statistically significant.

In order to further explore the different response between MDA-MB-231 and MCF-7 cells to Cy-3-glu, we mined the database (GEO34987) and screened out the top ten growth-related genes in MDA-MB-231 and MCF-7 cells (Table 1). Among them, we found that EGFR expression is 1.55 folds higher in MDA-MB-231 cells compared to MCF-7 cells. As the aberrant activation and over expression of EGFR leads to the dysregulation of signaling functions in cell proliferation, survival and cancer progression [29]. In addition, we additionally evaluated the expression profiles of ERα36 and EGFR in several BC cell lines from different clinical subtypes. We found a correlation between the expression of ERα36 and EGFR in the TNBC cell lines MDA-MB-231, MDA-MB-436 and BT20. In comparison, MCF-7 cells had lower level of ERα36 and nearly no expression of EGFR. Interestingly, SK-BR-3 cells express both ERα36 and EGFR but were insensitive to Cy-3-glu (Figure 1A, Figure 1B and Figure 7A). This could be explained by the over expression of HER-2 that is involved in some counteractive mechanisms in SK-BR-3 cells. Furthermore, we found that Cy-3-glu treatment dramatically decreased EGFR protein expression in MDA-MB-231 cells in a time-dependent manner (Figure 7B). However, no significant inhibition of EGFR expression at the mRNA level was detected after Cy-3-glu treatment (Figure 7C). It is well known that the intracellular degradation of proteins always takes place in the proteasome [30, 31]. As expected, the proteasome inhibitor MG132 completely restored the EGFR protein expression level (Figure 7D). Thus, promotion of EGFR degradation through the proteasome system was involved in the inhibition of TNBC cells growth mediated by Cy-3-glu.

**Table 1:** Growth-related genes (top 10) expression analysis of MDA-MB-231 vs. MCF-7 mined from the NCBI database (GSE34987)

Gene name	Fold increase in MDA-MB-231 vs. MCF-7 (mean ± SEM)
GPR116	2.05 ± 0.00
EMP1	1.92 ± 2.00
ADORA2B	1.64 ± 1.00
PDGFC	1.61 ± 1.00
EGFR	1.55 ± 1.00
CSF1R	1.54 ± 0.00
IGF2BP3	1.49 ± 0.00
CSF2	1.41 ± 0.00
PKIB	0.65 ± 1.00
GREB1	0.60 ± 0.00

GPR116: G protein-coupled receptor 116; EMP1: epithelial membrane protein 1; ADORA2B: adenosine A2b receptor; PDGFC: platelet derived growth factor C; EGFR: epidermal growth factor receptor; CSF1R: colony stimulating factor 1 receptor; IGF2BP3: insulin-like growth factor 2 mRNA binding protein 3; CSF2: colony stimulating factor 2 (granulocyte-macrophage); PKIB: protein kinase (cAMP-dependent, catalytic) inhibitor beta; GREB1: growth regulation by estrogen in breast cancer 1.

### Cy-3-glu inhibits the growth of MDA-MB-231 cells in vivo

**Figure 8:**
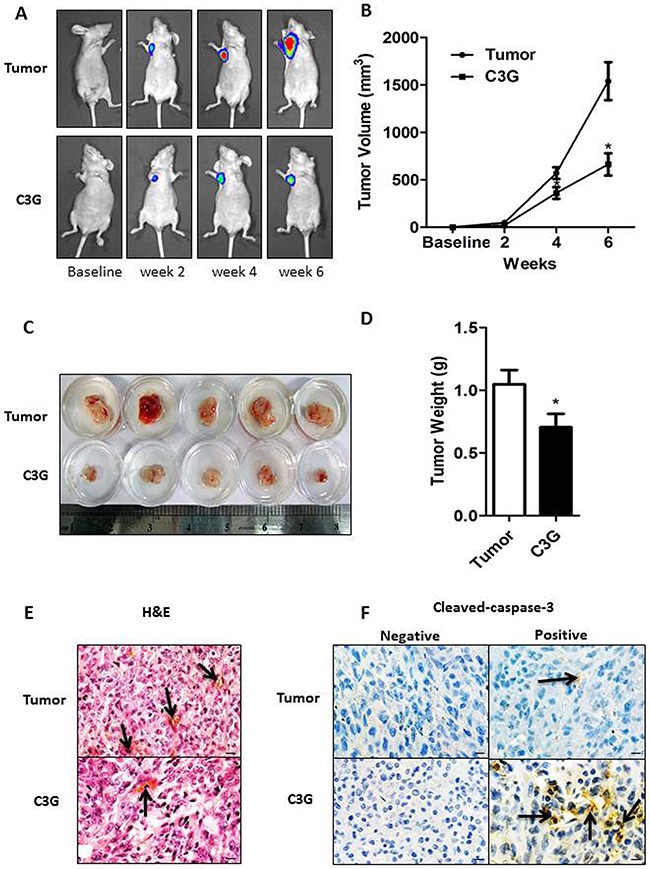
Cy-3-glu inhibits the growth of MDA-MB-231 cells in vivo In vivo tumor growth monitored by Xenogen IVIS imaging at different time points after MDA-MB-231-luc tumor implantation in female BALB/c athymic mice (n = 10) fed a control or Cy-3-glu diet for 6 weeks. **A.** orthotopic breast tumor growth was monitored in real time by bioluminescent imaging of luciferase activity in living mice using the cryogenically cooled IVIS-imaging system from baseline to week 6 post implantation. **B.** tumor growth was monitored (by a Vernier caliper) and presented as tumor volume in cubic millimeters, over a period of 6 weeks. **C** and **D.** photographic images of excised tumors were captured and its graphical representation of tumor weight (n = 5). **E.** hematoxylin and eosin staining of tumor, and capillaries are red (arrows). **F.** cleaved-caspase-3 is brownish in the cytoplasm (arrows) of tumor and Cy-3-glu-treated mice tumors. Differences with p < 0.05 (*), p < 0.01 (**) or p < 0.001(***) are considered statistically significant. Scale bar is 100 μm.

To examine the in vivo anticancer efficacy of Cy-3-glu, human breast tumor xenografts from athymic nude mice were used. By using a stably expressing luciferase (MDA-MB-231-luc) cell line to visualize and monitor the growth of BC in real-time, we observed that the tumor growth and volume in animals treated with Cy-3-glu were significantly smaller compared to the untreated control mice at the different time points evaluated (Figure 8A and 8B). The reduced tumor size and weight were further confirmed upon the scarification of mice (Figure 8C and 8D). Histological analysis showed that Cy-3-glu treatment significantly inhibited capillary formation (Figure 8E). The MVD was 25±1.66/mm^2^ (tumor group) vs. 4±0.33/mm^2^ (Cy-3-glu group). There were no differences in spleen and liver weights between the vehicle and Cy-3-glu-treated groups, which further revealed that Cy-3-glu has no toxicity (Supplementary Figure S5A and S5B). Body weights and food intake were monitored weekly as an indicator of overall health, and there were no differences between the control, tumor and Cy-3-glu-treated groups (Supplementary Figure S5C and S5D). Consistent with the in vitro experiments, apoptosis (as indicated by cleaved-caspase-3 staining) of the tumor specimen was significantly increased in the Cy-3-glu-treated group (Figure 8F). These results illustrated that Cy-3-glu also inhibits TNBC growth in vivo.

## DISCUSSION

**Figure 9:**
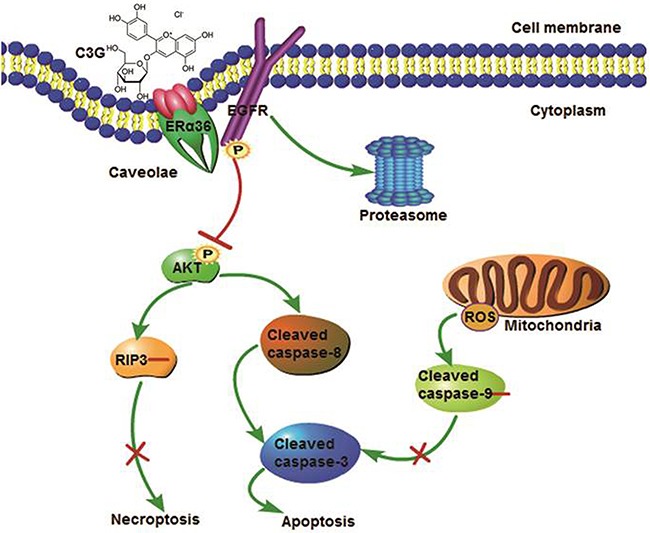
Putative mechanisms of the effects of Cy-3-glu action in the prevention of TNBC Cy-3-glu binds directly to the LBD of ERα36 and then inhibits its downstream EGFR/AKT signaling and promotes EGFR degradation through the proteasome system, which in turn drives TNBC cells apoptosis, independent of the mitochondrial intrinsic pathway.

The present study demonstrated that Cy-3-glu effectively promotes apoptotic cell death in a subset of TNBC cells that co-expressed ERα36 and EGFR. Mechanically, although ERα36 has not trans-membrane domain, it localizes to the caveolae of the plasma membrane invaginations. Cy-3-glu directly binds to the LBD of ERα36 and leads to the inhibition of its downstream EGFR/AKT signaling and promotes EGFR degradation through the proteasome system. This, in turn, drives the apoptosis of TNBC cells in a manner that is independent of the mitochondrial intrinsic pathway (Figure 9).

We observed the most significant inhibitory effect of Cy-3-glu on MDA-MB-231 cells among the three TN cell lines (MDA-MB-231, MDA-MB-436 and BT20). The expression of p53 was barely detectable in MDA-MB-436 cell line, whereas the overexpressed p53 were observed in MDA-MB-231 and BT20 cell lines. Moreover, as no expression of tumor suppressor protein RB1 in MDA-MB-436 cell line, normal expression level in BT20 cell line, and overexpression in MDA-MB-231 cell line [32], the different responses from cell lines are mainly due to their different gene mutations and expressions.

It has previously been reported that Cy-3-glu increases the proliferation of MDA-MB-231 by protecting the cells against the acrylamide-induced oxidative stress [14]. We observed the inhibitory effects of lower dose Cy-3-glu (5 and 10 μM) on MDA-MB-231 for 7 day and higher doses Cy-3-glu (150 and 500 μM) at 24 h and 48 h. The reason for this discrepancy may be the difference between the two studies in the treatment times of Cy-3-glu on MDA-MB-231 cells. They assessed the proliferation index at 4 h after the treatment, which is not a sufficiently long time for a cell cycle to occur. They also detected the inhibition of the production of ROS by Cy-3-glu after 4 h, which must represent the acute phase of the cells response upon the stimulation rather than the stable effects of the Cy-3-glu. We also evaluated the anti-oxidant activities of Cy-3-glu, but the activities of SOD and the production of mitochondrial ROS exhibited no significant difference between treatment with Cy-3-glu and vehicle DMSO in MDA-MB-231 cells. Accordingly, our result indicates that the main function of Cy-3-glu is to induce MDA-MB-231 cells apoptosis rather than to act as an anti-oxidant. A deliberate study showed that whole blueberry extracts inhibited the growth and metastatic potential of MDA-MB-231 cells [33, 34]. However, the precise component in the mechanisms underlying the anti-TNBC activity remains to be defined. In this study, we determined that the anthocyanin, Cy-3-glu, is an effective agent against TNBC. The preventive and therapeutic efficacy of Cy-3-glu against TNBC needs to be further studied in a large clinical investigation.

Our results shown Cy-3-glu could bind to LBD of ERα36, and has no binding affinity to the LBDs of ERα66 and ERα46. Compare to ERα66 and ERα46, ERα36 retains the partial LBD and possesses an extra, unique domain of 27 amino acids to replace the last 138 amino acids encoded by exons 7 and 8. The truncated LBD of ERα36 suggests that it may have a spectrum of ligand selectivity different from ERα66 and ERα46 [35]. In addition, although ERα36 has not trans-membrane domain, it localizes to the caveolae of the plasma membrane invaginations [36]. We and other groups have developed an affinity-purified rabbit polyclonal and monoclonal anti-ERα36 antibodies against 20 amino acids of the C-terminal of ERα36 [10, 36], and demonstrated that ERα36 promoted non-genomic estrogen signaling and cell growth could be blocked by anti-ERα36 antibody [36–38]. All these results indicated that ERα36 can receive the extracellular molecule signal. Additionally, the glycoside moiety will confer a hydrophilic nature to Cy-3-glu, but the other moiety of Cy-3-glu is an alkyl chain. Accordingly, it could be claimed that Cy-3-glu adsorbed to the cell membrane by the strength of electrostatic interactions of the polar groups of phospholipids and hydrophobic interactions with their alkyl chains.

Programed cell death has been reported to link three major routes, including apoptosis, necroptosis and autophagy [39, 40]. In particular, apoptosis and necroptosis are the two major modes of BC cell death [41, 42]. The determination for cells to undergo necroptosis or not is mediated by the factor RIP3 [43]. Since many cells do not express RIP3 [44], in this study we demonstrated that RIP3 was hardly detected in MDA-MB-231 cells. Thus, the Cy-3-glu induced MDA-MB-231 cell death was not through necroptosis. On the other hand, autophagy is always activated in response to metabolic stress, growth factor deprivation, limitation of nutrients and energy metabolism, or induced as an adaptive response against endoplasmic reticulum stress [45], which cannot induce the caspases cascade by TNF-α.

In conclusion, our data revealed that Cy-3-glu directly binds to ERα36, inhibits EGFR/AKT signaling, and also promotes EGFR degradation in TNBC. Even though the Cy-3-glu concentration is high and the translational application of Cy-3-glu itself is somehow limited, our study provides an insight into the mechanism whereby the natural compounds from plants would exert potential preventive or therapeutic effects in the treatments against TNBC.

## MATERIALS AND METHODS

### Compounds and antibodies

Cy-3-glu was purchased from Polyphenols AS Laboratories (Hanabryggene Technology Centre, Norway). The monoclonal and polyclonal antibodies for phospho-EGFR (Tyr845) (#6963), EGFR (#4267), phospho-AKT (Ser473) (#4060), cleaved-caspase-3 (#9664), caspase-3 (#9665), caspase-8 (#9746) and caspase-9 (#9508) were obtained from Cell Signaling Technology (CST, USA) and RIP3 was obtained from Abcam (ab16090, Abcam, US). The monoclonal and polyclonal antibodies for β-actin (sc-47778), AKT (sc-1619) and PCNA (sc-7907) were purchased from Santa Cruz Biotechnology, Inc. (Santa Cruz, USA). Anti-mouse or anti-rabbit secondary horseradish peroxidase (HRP) conjugate was obtained from ZSGB-BIO (Beijing, China). 17β-estradiol (E2), Dimethylsulfoxide (DMSO), phosphate buffered saline (PBS), and other chemicals were purchased from Sigma-Aldrich (Sigma, USA). SNG162, an ERα36 specific inhibitor, the monoclonal antibody of ERα36 and ERα36 ligand binding domain fused with Fc (Fc-LBD) protein were kindly provided by Shenogen Pharma Group Inc. (Beijing, China).

### Cell cultures and treatments

The human ER and PR positive BC cell line MCF-7 (ATCC® HTB-22™), HER-2 positive BC cell line SK-BR-3 (ATCC® HTB-30™), TNBC cell lines MDA-MB-436 (ATCC® HTB-130™), BT-20 (ATCC® HTB-19™) and MDA-MB-231 (ATCC® CRM-HTB-26™), and human non-cancerous mammary epithelial cell line (MCF-10A; ATCC® CRL-10317™) were purchased from the American Type Culture Collection (Manassas, USA). MDA-MB-231-luc cell line was a gift by Dr. Bin Gao, Ph.D., Professor from Chinese Academy of Sciences. MCF-7, SK-BR-3 and BT20 cells were maintained in Dulbecco's modified Eagle's medium (DMEM; Sigma, USA) supplemented with 10% FBS, 100 U/mL penicillin and 50 U/mL streptomycin in a humidified atmosphere containing 5% CO_2_, at 37°C. MDA-MB-231 and MDA-MB-436 cells were maintained in L15 medium (Sigma, USA) supplemented with 10% FBS, 100 U/mL penicillin and 50 U/mL streptomycin in a humidified atmosphere at 37°C. MCF-10A cells were cultured in DMEM/Nutrient Mixture F-12 Ham medium (DMEM/F-12; Sigma, USA) containing 5% horse serum in the presence of 10 μg/mL insulin (Sigma, USA), 20 ng/mL epidermal growth factor (EGF; Sigma, USA), 100 ng/mL cholera toxins (Sigma, USA), 0.5 μg/mL hydrocortisone (Sigma, USA), 100 U/mL penicillin, and 50 U/mL streptomycin. All cells were used in experiments during the linear phase of growth.

### Cell viability and cytotoxicity assay

Cells were suspended at a final concentration of 4 × 10^3^ cells/well in 96-well flat-bottomed micro plates. Cells at 60–75% confluence were treated with Cy-3-glu. After removing the culture supernatant, CCK-8 (10 μL) was added to each well containing 100 μL of culture medium and the plate was incubated for 2 h at 37 °C. Viable cells were assessed by absorbance measurements at 450 nm using a monochromatormicroplate reader (BioTek, USA). For cytotoxicity assay: measurement of the cell membrane integrity is a parameter for the cell death. Briefly, 2× CellTox Green Reagent (Promega, USA) included in each well at the time of Cy-3-glu dosing, and the provided lysis solution at 1:25 ratio was used as a positive control. Fluorescence was measured using a Tecan Infinite plate reader with 485–510 nm excitation and 520–530 nm emission. All experiments were performed in triplicate on three separate occasions and the data are presented as the mean of the triplicate.

### Cell cycle analysis

Cells were collected and washed in cold PBS, and fixed gently in 70% ethanol overnight at 4°C. After resuspension in PBS containing 0.01 mg/mL propidium iodide (PI; Sigma, USA) and 0.1 mg/mL RNase, cells were incubated in the dark for 30 min and the stained cells were analyzed with a FC-500 flow cytometer (Beckman Coulter, USA). Cell cycle distribution was analyzed using MultiCycle software (Phoenix Flow Systems, USA). For each experiment, 10,000 cells were recorded. Each experiment was run in triplicate and carried out three individual times.

### Apoptosis assay

Cells were analyzed by Flow cytometer analysis. Briefly, cells treated with DMSO or Cy-3-glu were trypsinized, washed in cold PBS, dual stained with the Annexin V/PI apoptosis detection kit (Sigma, USA) following the manufacturer's instructions and signals were detected through FL-1 (FITC) and FL-3 (PI) detectors. For each analysis, 5,000-10000 cells were recorded.

### TUNEL staining assay

Apoptotic cell death was confirmed by the terminal deoxynucleotidyltransferase-mediated dUTP nick end-labeling (TUNEL) technique as described by the In Situ Cell Death Detection kit, POD (Roche, Germany) for DNA chromatin morphologic features used during quantification following the manufacturer's guidelines. For quantification of apoptosis, the results were viewed under a fluorescence microscope (Olympus, Japan). At least 1000 cells from more than ten random microscopic fields were counted by two observers. We quantified TUNEL-positive staining cells by counting cells in 20 fields of each slide at an original magnification of ×200 using NIH Image J 1.42 program (http://rsb.info.nih.gov/ij/download.html). For flow cytometry: cells were fixed with 4% paraformaldehyde and then cells were resuspended in 0.1% Triton X-100 permeabilization solution, subjected to TUNEL staining, and analysed by flow cytometry using a FC-500 flow cytometer (Beckman Coulter, USA).

### Measurement of mitochondrial ROS

Cells treated with DMSO or Cy-3-glu were removed from the culture medium at 24 h and stained with MitoTrackerRed CM-H2XRos (Invitrogen, USA) at 37°C in a humidified 5% CO_2_ atmosphere for 30 min. Cells were then collected with trypsin, washed in cold PBS, and analyzed on a flow cytometer (Beckman Coulter, USA).

### SOD activity assay

Cells treated with DMSO or Cy-3-glu for 24 h and cell extracts were prepared and protein concentration was determined by the Bio-Rad protein assay kit (cat. 500-0002; BioRad, USA). SOD activity was performed according to the instruction of the assay kit (Beyotime, China). All experiments were performed in triplicate in three individual times, and the data are presented as the mean of the triplicate. The SOD activity was expressed on mg protein.

### RNA extraction and real-time RT-PCR

Total RNA and quantitative real-time PCR (qPCR) were performed as described previously [46]. Primer pairs are shown in Table 2. Data were analyzed with LightCycler® 480 software, Version 1.5 (Roche). Relative quantification of gene expression was performed using standard curves and normalized to the value for glyceraldehyde 3-phosphate dehydrogenase (GAPDH) in each sample. The films were scanned and quantified using the NIH Image J 1.42 program (http://rsb.info.nih.gov/ij/download.html), each experiment was run in triplicate.

**Table 2:** The sequences of the oligonucleotide primers and amplified products of real-time PCR

Gene name	Sequence of primer (5′–3′)	Amplified product (bp)
GAPDH	F: ATGGGGAAGGTGAAGGTCG	107
	R: GGGGTCATTGATGGCAACAATA	
Cyclin D1	F: ACCTGAGGAGCCCCAACAA	105
	R: TCTGCTCCTGGCAGGCC	
Cyclin B1	F: TGGACTATGACATGGTGCACTT	156
	R: GCCAGGTGCTGCATAACTG	
Cyclin E	F: CGCCTGCCGGGACTGGAG	110
	R: TCTTCCTGGAGCGAGCCG	
CDK2	F: CCCTTTCTTCCAGGATGTGA	134
	R: TGAGTCCAAATAGCCCAAGG	
CDK4	F: GAAACTCTGAAGCCGACCAG	78
	R: ACATCTCGAGGCCAGTCATC	
CDK6	F: TCCCTCCTTTGAAGTGGATG	148
	R: GTCACCTGGGGCTAAATGAA	
EGFR	F: AGGCACGAGTAACAAGCTCAC	176
	R: ATGAGGACATAACCAGCCACC	

F: forward; R: reverse; GAPDH: glyceraldehyde 3-phosphate dehydrogenase; CDK2: cyclin-dependent kinase 2; CDK4: cyclin-dependent kinase 4; CDK6: cyclin-dependent kinase 6; EGFR: epidermal growth factor receptor.

### Western blotting

Protein was extracted from cell samples using the RIPA method and quantified by a Bio-Rad protein assay kit (cat. 500-0002; BioRad, USA). Protein samples were separated by SDS-PAGE and then transferred to a PVDF membrane (cat. IPVH00010; Millipore, USA). The membrane was incubated overnight at 4°C with the primary antibody diluted in 5% non-fat dry milk. The membranes were washed 3 times and incubated for 45 min at room temperature with the HRP conjugated secondary antibody (Beijing, China). Each reaction was performed in triplicate in three independent Western blotting assays. The films were scanned and quantified using the NIH Image J 1.42 program (http://rsb.info.nih.gov/ij/download.html).

### In vivo tumor growth and bioluminescent imaging

Five-week-old intact female BALB/c athymic mice were purchased (Animal Center, Academy of Military Medical Sciences, China) and randomly divided into three groups (n = 10 for each group). The control group and tumor model group were fed a normal diet (ND; n = 10) (Supplementary Table S2), and the Cy-3-glu group was fed ND containing Cy-3-glu 420 mg/kg ND (n = 10). At 6 weeks of age, mice were injected s.c. with MDA-MB-231-luc cells (2 × 10^6^) in matrigel (BD Biosciences, USA). Body weights and food intake were monitored weekly. Tumor growth was monitored in real time with bioluminescent imaging of luciferase activity in live mice using the cryogenically cooled IVIS-imaging system (Calipers, USA). Tumor size was measured weekly using calipers, and the volume was calculated with the formula [(4/3πr_1_^2^×r_2_) (0.125)], where r_1_ is the smaller radius and r_2_ is the larger radius. At 12 weeks of age, mice were sacrificed. All animal experiments were performed in compliance with the Institutional Animal Care and Use Committee guidelines of CAU and the permission number was SKLAB-2014-01-05. Four weeks post injection, mice were euthanized and tumors were removed, weighed, and identified with hematoxylin and eosin staining and cleaved-caspase-3 histological staining.

### Expression and purification of LBD-ERα66

The human LBD-ERα66 was expressed by PET30a vector. The inclusion bodies of bacterial pellet were resolved in buffer containing 50 mM Tris-HCL (pH 8.0), 100 mM NaCl, 10 mM EDTA, 6 M guanidine hydrochloride, 10% glycerin and 10 mM DTT. After then inclusion bodies were diluted into refolding buffer (100 mM Tris [pH 8.5], 400 mM L-Arg HCl, 2 mM EDTA, 5 mM reduced glutathione and 0.5 mM oxidized glutathione) and stirred overnight at 4°C. Subsequently, the refolded protein was concentrated to less than 50 mL and exchanged into buffer containing 20 mM Tris-HCL (pH 8.5), 150 mM NaCl, 2 mM EDTA and 2 mM DTT. Finally 2 mL sample was further purified by gel filtration chromatography (Superdex-20016/60 GL column, GE Healthcare).

### The quantification of microvasculature density

Microvessel density (MVD) was quantified according to the methods established by Weidner [47]. Briefly, all section slides were scanned at low power (×100) to identify the “hot spots”, which represented the areas of highest neovascularization. The individual microvessels were later counted under high power (×400) to obtain a vessel count in a defined area. The average vessel count for the five “hot spots” was calculated as the MVD. The results were calculated as endometrial MVD/mm^2^ (vessels/mm^2^). Differences with p < 0.01 (**), n = 20 fields compared.

### Immunohistochemistry

This method was performed as previously described [48]. Briefly, four micron tissue sections were deparaffinized in xylene and rehydrated through a series of decreasing ethanol concentrations. The slides were pretreated with hydrogen peroxide (3%) for 10 min to remove endogenous peroxidase, followed by antigen retrieval in a microwave for 15 min in 10 mM citrate buffer (pH 6.0). The primary antibodies were applied, followed by washing and incubation with the biotinylated secondary antibody for 30 min at room temperature. The slides were counterstained with hematoxylin and dehydrated in alcohol and xylene before mounting.

### Microscale thermophoretic analysis

MicroScale Thermophoresis (MST) was used to study molecular interactions in solution [49, 50]. The binding affinity of Cy-3-glu with the purified ERα36 ligand binding domain (LBD) and E2 with LBD-ERα66 proteins were measured by MST analysis (Monolith NT. 115, NanoTemper Technologies GmbH, Germany), E2 served as a positive control for ERα66 LBD binding assay. Briefly, the LBD proteins were labeled with a Cy5/Alexa 647 fluorescence dye (NT647, NanoTemper Technologies GmbH, Germany). Cy-3-glu was titrated between 0.61 and 2500 μM and E2 was between 0.49 and 1000 nM to a constant amount of purified ERα36 or ERα66 LBD proteins (100 nM). Data were analyzed using NTAnalysis software. Each reaction was performed in triplicate for three independent assays.

### Knocked down ERα36 in MDA-MB-231 cells

MDA-MB-231 cells with ERα36 expression knocked down by the shRNA method was established previously [51]. Briefly, MDA-MB-231 cells were transfected with the empty expression vector or the ERα36-specific shRNA expression vector pRNAT-U6.1/Neo plasmid containing the shRNA against ERα36 (GenScript, USA) with Lipofectamine 3000 according to the manufacturer's instruction. Forty-eight hours after transfection, the cells were re-plated and selected with G418 (300 mg/mL) for two weeks. Clones were expanded for further analysis.

### Microarray data

A list of growth-related genes (top 10) in MDA-MB-231 cells compared MCF-7 cells were mined from the GEO data set GSE34987 (http://www.ncbi.nlm.nih.gov/geo/geo2r/?acc=GSE34987) [52]. Top ten candidates were obtained from the mined NCBI database with the GEO2R module.

### Statistical analysis

One-way ANOVA, Dunnett's post hoc tests, Spearman and Kendall's tau-b tests were performed for statistical analyses using the SPSS18.0.1 Package (SPSS Inc., Chicago, IL). Differences with p < 0.05 (*), p< 0.01 (**) or p< 0.001(***) was considered statistically significant. Values are presented as the mean ± SEM (standard error of mean).

## SUPPLEMENTARY MATERIALS FIGURES AND TABLES


