# Supplementary material for: Cyanidin-3-o-glucoside directly binds to ERα36 and inhibits EGFR-positive triple-negative breast cancer
Source: Oncotarget. 2016 Sep 15;7(42):68864–82. doi: 10.18632/oncotarget.12025 (PMC5356596; doi:10.18632/oncotarget.12025)
Supplement: Supplementary file 1 [file oncotarget-07-68864-s001.pdf]

Cyanidin-3-o-glucoside directly binds to ERα36 and inhibits EGFR-positive triple-negative breast cancer

SUPPLEMENTARY FIGURES AND TABLES

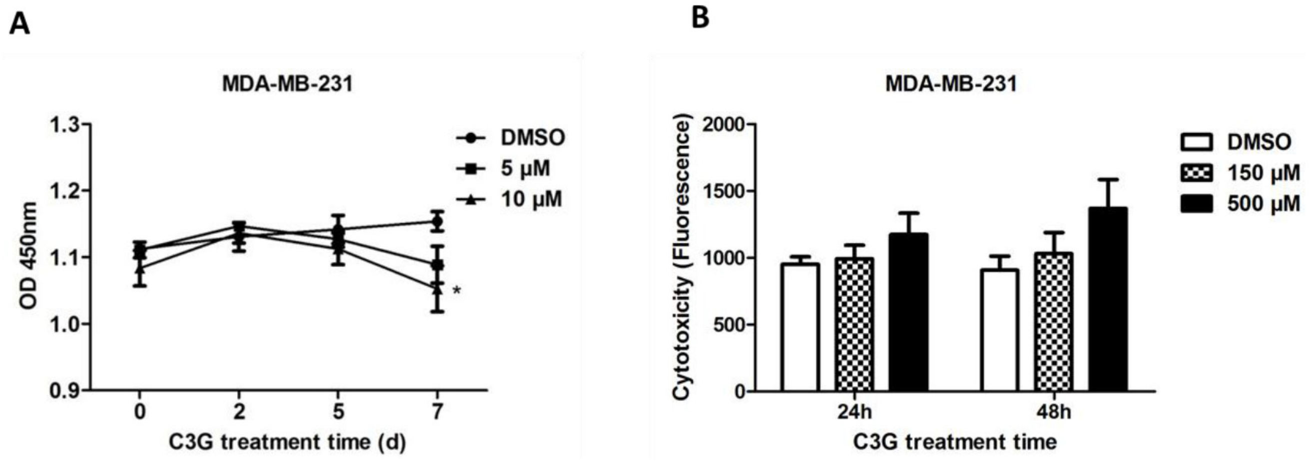

Supplementary Figure S1: The effect of Cy-3-glu on the growth and cytotoxicity in MDA-MB-231 cells. A. MDA-MB-231 cells were treated with 5 μM and 10 μM Cy-3-glu for 7 d, and DMSO serves as the vehicle control. **B.** the cytotoxicity of higher dose (150 and 500 μM) of Cy-3-glu in MDA-MB-231 cells. The results represent the mean ± SEM from three independent experiments.

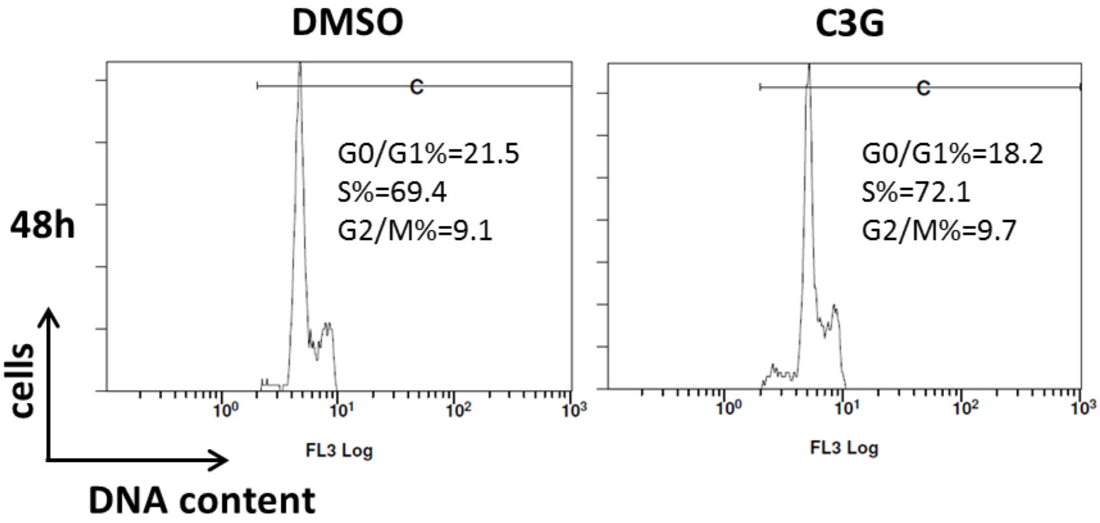

Supplementary Figure S2: The effect of Cy-3-glu on cell-cycle at 48 h in MDA-MB-231 cells. DNA content was measured by PI staining (x axis) and the population of cells was measured (y axis), the pre-G1 region indicated the apoptosis. Triplicate measurements were performed for each experiment.

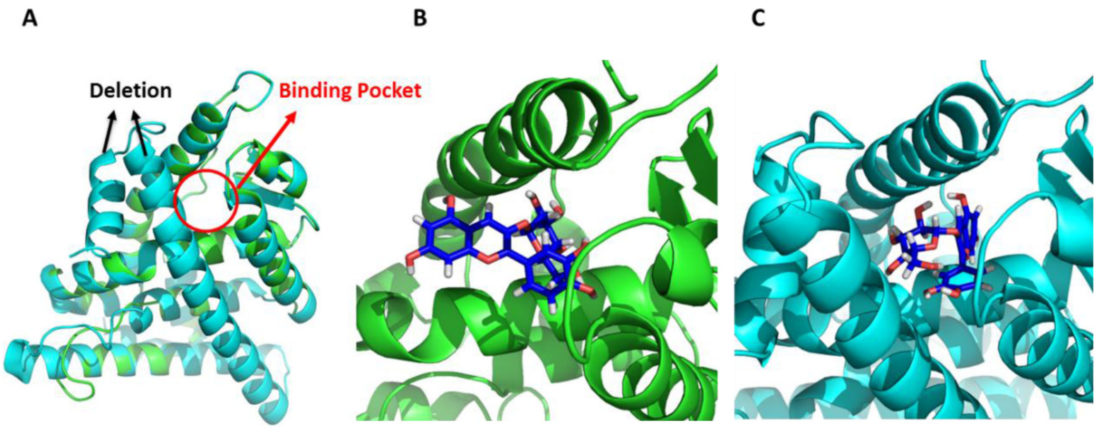

Supplementary Figure S3: The conformation of LBD of ERα36 and ERα66 and their molecular docking with Cy-3-glu by Auto Dock program. A. merging conformations of LBD-ERα36 (green) and LBD-ERα66 (light blue). **B.** molecular docking of Cy-3-glu (dark blue) with LBD-ERα36 (green) and the binding energy is -79.89 kcal/mol. **C.** molecular docking of Cy-3-glu (dark blue) with LBD-ERα66 (light blue) and the binding energy is -28.27 kcal/mol.

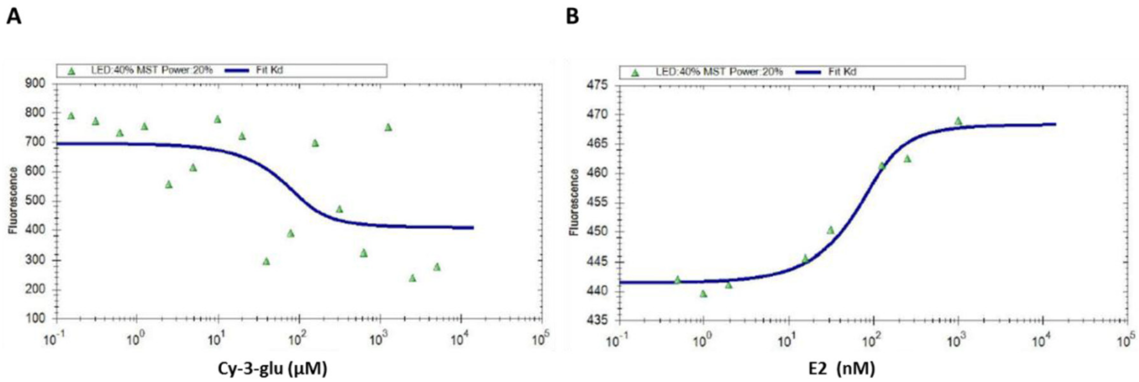

Supplementary Figure S4: The binding analysis of LBD-ERα66 to Cy-3-glu and E2. A. microscale thermophoresis analysis of Cy-3-glu binding to LBD-ERα66, no binding was observed. **B.** microscale thermophoresis analysis of E2 binding to LBD-ERα66 with a KD of 21.3 ± 1.25 nM. Triplicate measurements were performed for each experiment.

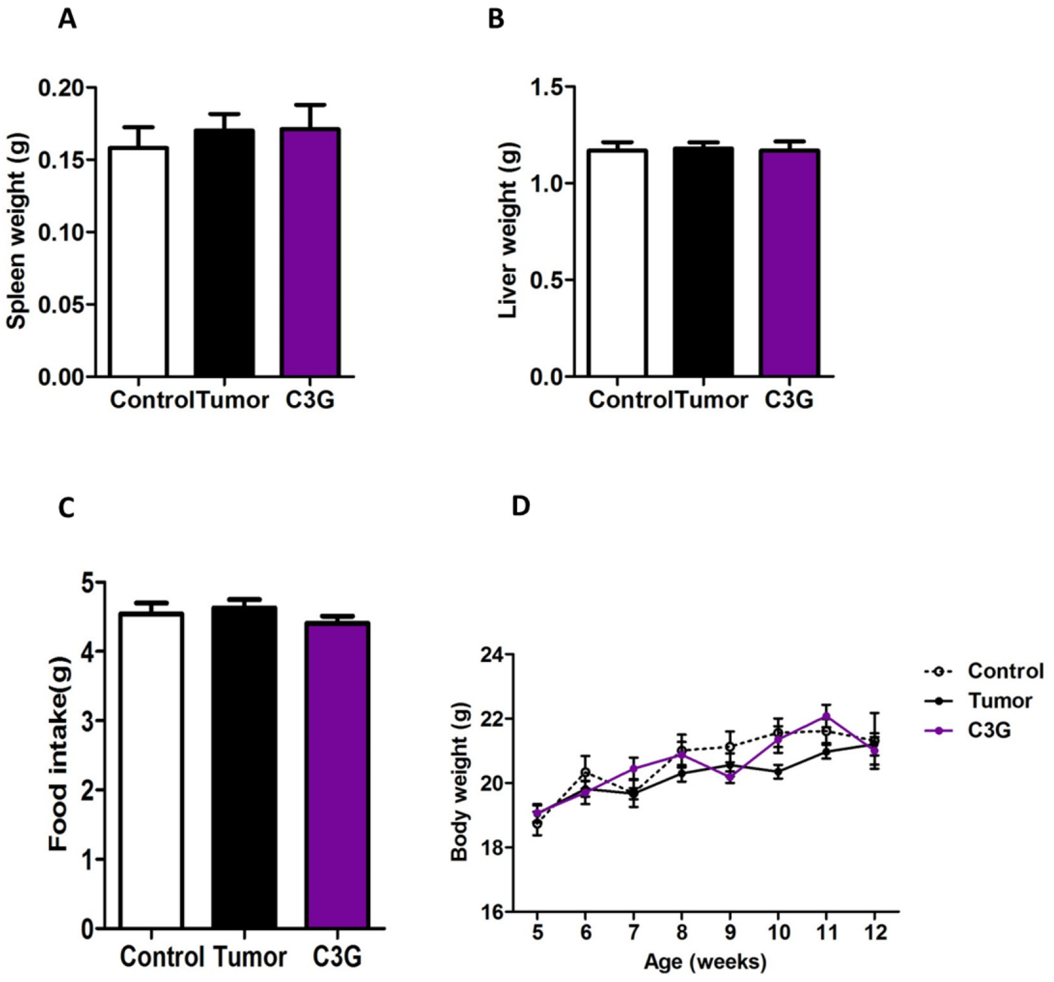

Supplementary Figure S5: Characterization of MDA-MB-231 cells in vivo. A. spleen weigh (g) of group of control, tumor and Cy-3-glu, n = 10 per group. **B.** liver weigh (g) of group of control, tumor and Cy-3-glu, n = 10 per group. **C.** Daily food intake (g) of group of control, tumor and Cy-3-glu, n = 10 per group. Control and tumor group fed normal diet, Cy-3-glu group fed Cy-3-glu diet. **D.** Body weight gain of group of control, tumor and Cy-3-glu, n = 10 per group. Data are mean ± SEM.

Supplementary Table S1: Glycosidase class of anthocyanidins contents of common garden stuff

common garden stuff	cy-3-glu	dp-3-glu	pt-3-glu	pn-3-glu	pg-3-glu	mv-3-glu
Cranberry	7.4-39.6			40.4		
Black Currant	124.8-285.6	270-1132.1				
Gooseberry	0.49-54.2			0.5-1.3		
Chokeberry	16.9-376					
Elderberry	2141-7398				18	
Tart cherries	5.2-27.6					
Sweet Cherry	22.6-441			1.4-7.6	2	
Black Raspberry	359-890					
Purple basil	3.8					
Mashua Potato	7.3-47.5	7.4-35.6				
Red Raspberry	29-251.2	28.8			26.7-42.3	28.5-36.4
Blackberry	640-1532.7					
Plum	79.3-7590			60-1190		
BayLaurel berries	90000			10600		

values are listed in µg/g and anthocyanin concentrations may vary within a plant species due to variety, conditions under which the product was sampled and even methods of calculation. Anthocyanin abbreviations are as follows: cy, cyanidin; dp, delphinidin; pt, petunidin; pn, peonidin; pg, pelargonidin; mv, malvidin; glu, glucoside.

Supplementary Table S2: Normal diet (ND) information

moisture % ≤ 8.0
Protein % ≥ 18.0
Fat % ≥ 4.0
Fiber % ≤ 5.0
Ash % ≤ 6.5
Calcium % 1.2 ~ 1.4
phosphorus % 0.8 ~ 1.0
Magnesium % ≥ 0.2
potassium % ≥ 0.5
Sodium % ≥ 0.2
Iron, mg/Kg ≥ 150.0
Manganese, mg/Kg ≥ 75.0
Cuprum, mg/Kg ≥ 15.0
Zinc, mg/Kg ≥ 60.0
Iodine, mg/Kg ≥ 0.5
Selenium, mg/Kg 0.1 ~ 0.2
